# Optimization of Microwave Assisted Extraction Conditions to Improve Phenolic Content and In Vitro Antioxidant and Anti-Microbial Activity in *Quercus cerris* Bark Extracts

**DOI:** 10.3390/plants11030240

**Published:** 2022-01-18

**Authors:** Adrian Nisca, Ruxandra Ștefănescu, Cadmiel Moldovan, Andrei Mocan, Anca Delia Mare, Cristina Nicoleta Ciurea, Adrian Man, Daniela-Lucia Muntean, Corneliu Tanase

**Affiliations:** 1 Doctoral School of Medicine and Pharmacy, “George Emil Palade” University of Medicine, Pharmacy, Sciences and Technology of Târgu Mureș, 38 Gheorghe Marinescu Street, 540139 Târgu Mureș, Romania; adrian.nisca@umfst.ro; 2 Department of Pharmacognosy and Phytotherapy, Faculty of Pharmacy, “George Emil Palade” University of Medicine, Pharmacy, Sciences and Technology of Târgu Mureș, 38 Gheorghe Marinescu Street, 540139 Târgu Mureș, Romania; 3 Department of Pharmaceutical Botany, Faculty of Pharmacy, “Iuliu Hațieganu” University of Medicine and Pharmacy, 8 Victor Babeș Street, 400012 Cluj-Napoca, Romania; cadmiel.moldovan@umfcluj.ro (C.M.); mocan.andrei@umfcluj.ro (A.M.); 4 Laboratory of Chromatography, Institute of Advanced Horticulture Research of Transylvania, University of Agricultural Sciences and Veterinary Medicine, 400372 Cluj-Napoca, Romania; 5 Department of Microbiology, Faculty of Medicine, “George Emil Palade” University of Medicine, Pharmacy, Sciences and Technology of Târgu Mureș, 38 Gheorghe Marinescu Street, 540139 Târgu Mureș, Romania; anca.mare@umfst.ro (A.D.M.); cristina.ciurea@umfst.ro (C.N.C.); adrian.man@umfst.ro (A.M.); 6 Department of Analytical Chemistry and Drug Analysis, Faculty of Pharmacy, University of Medicine, Pharmacy, Sciences and Technology of Târgu-Mureș, Gh. Marinescu Street No. 38, 540139 Târgu Mureș, Romania; daniela.muntean@umfst.ro; 7 Department of Pharmaceutical Botany, Faculty of Pharmacy, “George Emil Palade” University of Medicine, Pharmacy, Sciences and Technology of Târgu Mures, 38 Gheorghe Marinescu Street, 540139 Târgu Mures, Romania; corneliu.tanase@umfst.ro

**Keywords:** antibacterial activity, radical scavenge activity, Gram-positive, *Klebsiella pneumoniae*, optimization, polyphenols, *Quercus*, turkey oak

## Abstract

The species of the *Quercus* genus, including *Quercus cerris* L., are widely used and their wood represents a valuable material utilized for various purposes. The intense industrial processing of wood results in a considerable amount of poorly-used natural by-products, such as bark, and a loss of potentially useful raw materials. Thus, the aim of our study was to evaluate the phytochemical characteristics and potential biological activities of *Quercus cerris* bark extracts obtained by optimizing the parameters of microwave assisted extraction (MAE). The optimum conditions for MAE were determined using a design of experiments (DoE) model, which set the experimental variables (irradiation time and microwave power) and their values. Aqueous and hydroalcoholic extractions were performed and the optimum parameters of extraction were determined for both solvents. The total polyphenolic and tannin contents were determined. The biological activities representative of antioxidant capacity were determined using two free radical scavenging methods, the DPPH and ABTS methods, and the antibacterial activity was assessed with the microdilution method. The results showed different optimal extraction conditions for aqueous (30 min at 850 W) and hydroalcoholic (18 min at 650 W) extracts. A higher yield of total polyphenols was observed in the hydroalcoholic bark extract (403.73 ± 7.35 mg gallic acid equivalents/g dried weight); however a lower level of tannins was registered in comparison to the aqueous extract. In addition, both extracts exerted high antioxidant activities, with the aqueous extracts having a stronger inhibitory effect against the DPPH radical. Moreover, the extracts exhibited antibacterial activity against the tested bacterial strains, especially against the Gram-positive strains and *Klebsiella pneumoniae*, with the hydroalcoholic extracts being more efficient overall. To conclude, the optimized MAE was an efficient method to extract phytochemical compounds with potential biological effects from *Quercus cerris* bark.

## 1. Introduction

The *Quercus* genus is one of the most important genera of the Fagaceae family, as it comprises over 600 species, mainly trees, that are widely spread in the Northern Hemisphere, mainly in Europe, Asia, and the Americas (especially North America) [1,2]. Due to the properties of their wood, oaks have great economic importance, and they are used in different fields, such as construction, furniture, and barrel production [3,4]. Considering the current spread of the genus and the value of oak wood, the large amount of oak bark that results after wood processing is considered natural waste. However, recent research focused on the analysis of different types of oak barks suggested that this so-called natural waste might be an important source of bioactive compounds, mainly polyphenols, such as phenolic acids, tannins, and flavonoids [5]. It is believed that because of these secondary metabolites, the oak bark extracts can exert a series of biological activities, including antioxidant, antimicrobial, anti-inflammatory, antitumoral, cardioprotective, and hepatoprotective activities [6,7,8].

Turkey oak (*Quercus cerris* L.) represents one of the native oak species found in central Europe [4], and one of the oak species found in all the regions of Romania but mainly in the western part of the country [9,10,11]. Data regarding the phytochemical composition and the biological activity of the *Q. cerris* bark extracts are very limited. Previous studies focus more on the potential use of the bark as a material, due to some of the physical characteristics of the cork, rather than on the identification and use of the bioactive compounds that may be found in the vegetal matrix [12,13,14]. However, in one study, Sen et al. determined the total phenolic acids, condensed tannins, and flavonoid contents in hydroalcoholic extracts obtained from the cork and phloem fractions of *Q. cerris*. The same study indicated the antioxidant capacity of the extracts [15]. Additionally, phenolic compounds, such as vanillic, homovanillic, and isovanillic acids, vanillin, and syringic acid, were identified in the hydroalcoholic extracts obtained from the wood of *Q. cerris*. These extracts also exerted antioxidant activities [16]. The presence of these compounds in the wood may indicate their potential presence in the bark as well. Moreover, aqueous and methanolic *Q. cerris* bark extracts have been shown to inhibit the proliferation of the Hep-2 (human larynx carcinoma) cell line [17].

To benefit from the health-improving effects provided by the bioactive compounds comprised in vegetal matrices, such as bark, these phytochemicals must first be extracted using specific extraction methods (i.e., the Soxhlet method, ultrasound assisted, or microwave assisted extractions) [18,19]. Microwave assisted extraction (MAE) is one of the most modern and efficient methods used to recover bioactive compounds from vegetal matrices [20]; however, a prior optimization of the extraction parameters must be performed to assure that the maximum yield of phytochemical compounds is achieved [21].

To the best of our knowledge, there are no studies focused on the optimization of the MAE of phenol compounds from *Q. cerris* bark, and even less using a “green solvent” as water. Therefore, a systematic approach was used to optimize the extraction factors, including microwave power and irradiation time, leading to the maximum extraction of phenols and the highest in vitro antioxidant and antibacterial activity.

Thus, the aim of this study was to optimize the extraction parameters of microwave assisted extraction (used solvent, microwave power, and irradiation time) of *Q. cerris* bark and to determine the phytochemical profile and the potential biological activities of the extracts obtained via the optimized parameters.

## 2. Materials and Methods

### 2.1. Chemicals, Reagents, and Bacterial Strains

The chemicals used for the extraction procedures and total phenolic content determination were 95% ethanol (used to obtain a 70% solution) purchased from Girelli Alcool Srl (Zibido, San Giacomo, Italy), Na_2_CO_3_ decahydrate purchased from Reactivul Srl (Râmnicu, Vâlcea, Romania), gallic acid monohydrate purchased from Sigma-Aldrich Chemie GmbH (Steinheim, Germany), and Folin–Ciocâlteu reagent purchased from Merck KGaA (Darmstadt, Germany). The protocols used for the assessment of total tannin content and antioxidant activity additionally required 2,2-diphenyl-1-picrylhydrazyl (DPPH), 2,2′-azino-bis(3-ethylbenzothiazoline-6-sulfonic acid) (ABTS), hide powder, and pyrogallol, which were all acquired from Sigma-Aldrich Chemie GmbH (Steinheim, Germany).

The antibacterial activity was tested on 5 different bacterial strains: *Staphylococcus aureus* ATCC 25923, Methicillin-resistant *Staphylococcus aureus* ATCC 43300, *Escherichia coli* ATCC 25922, *Klebsiella pneumoniae* ATCC 13883, and *Pseudomonas aeruginosa* ATCC 27853. These strains were provided by the Microbiology Department of the George Emil Palade University of Medicine, Pharmacy, Sciences, and Technology from Târgu-Mureș.

### 2.2. Plant Sample

The *Quercus cerris* L. bark samples were collected from Deva, Hunedoara County, Romania, during May 2021. The bark was collected from the stems of *Q. cerris* specimens, aged between 15 and 20 years. These samples were collected by using the itinerary method. The samples were shredded manually and dried in a Nahita 631 Plus drying oven (Auxilab S.L., Beriáin, Spain) at 50 °C for 24 h. The dried material was then milled using a Pulverisette 15 cutting mill (Fritsch GmbH, Idar-Oberstein, Germany).

### 2.3. Optimization and Extraction/Design of Experiments (DoE)

Before the development of a design of experiments (DoE) that would allow the comprehensive study of the extraction process, some preliminary experiments were performed. The output of these experiments was analyzed by a team of experienced researchers. The aim of this was to define the most significant process parameters and their fundamental values (data not shown).

MODDE 13.1 software (Sartorius Stedim, Umeå, Sweden) was employed for the development of a D-optimal DoE, which permitted the integration of the experimental variability for studying its effects and established the optimal experimental values for the extraction.

The D-optimal model allows the study of multiple combinations of qualitative and quantitative multilevel factors in the same experimental design. Unlike standard classical designs, such as factorials and fractional factorials, D-optimal design matrices are usually not orthogonal and effect estimates are correlated. In this model, several experimental runs are chosen to include the largest possible volume of the variability matrix. [22].

Furthermore, by the analysis of coefficients, we evaluated the effects of the process variables over the measured extraction performances. Next, the optimal formulation and design space were defined in accordance with the obtained results.

A proven acceptable range (PAR) was established within the design space. The PAR is defined as “a characterized range of a process parameter for which operation within this range, while keeping other parameters constant, will result in producing a material meeting relevant quality criteria” [23]. After the determination of the design space and PAR, the optimal extraction parameters were established by defining combinations of factors (i.e., microwave power, irradiation time) that predicted a result as close as possible to the target values of the response [24].

The developed optimization DoE considered two quantitative factors: irradiation time, varied over 3 levels of variation (10, 20, and 30 min) and microwave power, varied over 3 levels of variation (200, 500, and 1000 W). It consisted in 14 experimental runs, including 3 replicates performed in order to assess the reproducibility of the extractions. To reduce the risk of systematic errors, the 14 runs were performed in a randomized order. These data are presented in Table 1.

Ten grams of *Q. cerris* bark were placed into the microwave extractor vessel with 200 mL of water or 70% ethanol. The extractions were performed using all the combinations of the above-mentioned parameters in an Ethos X Advanced microwave extractor (Milestone, Sorisole, Italy). All the extracts were filtered through vacuum filtration and then added to a 200 mL volumetric flask, completing the volume with the specific solvent. To evaluate the optimum parameters for both solvents used, the total phenolic content (TPC) of the extracts was determined. The results were expressed as mg gallic acid equivalents (GAE)/g dried bark. After determining the optimal conditions of extraction for both solvents, the extractions were reperformed with these parameters. The extracts obtained via the optimal parameters were centrifuged for 11 min at 15,000 RPM and 10 °C with a Hettich Mikro 200R (Andreas Hettich GmbH, Tuttlingen, Germany). The supernatant was carefully collected and reused in the next steps. The ethanolic extracts were then concentrated in a rotary evaporator at 75 °C, removing the ethanol; this step was required to freeze the extracts. Finally, the unconcentrated aqueous extracts and concentrated ethanolic extracts were freeze dried in a BK-FD12S freeze dryer (Biobase Biodustry Co., Ltd., Shandong, China).

### 2.4. Quantification of Total Phenolics

The total polyphenolic content was assessed using the Folin–Ciocâlteu method, as previously described with slight modifications [25].

The TPC of the aqueous (QCA) and hydroalcoholic (QCE) extracts obtained using the parameters set by the design of experiments software was determined by adding 400 µL of Folin–Ciocâlteu reagent to 400 µL of appropriately diluted extract, and finally adding 3200 µL of 5% Na_2_CO_3_ solution, shaking the test tubes well and leaving them at room temperature in darkness for one hour. For each diluted extract, 3 replicates were performed. The absorbance of each replicate was measured at 750 nm with a Specord 200 Plus (Analytik Jena AG, Jena, Germany). The results were expressed as mg GAE/g dried bark.

The TPC of the extracts obtained using the optimal parameters was determined before and after freeze drying. Approximately 2 mg of lyophilized extract were redissolved in 1 mL solvent used for extraction. The reconstituted solutions were then similarly analyzed. The results were expressed as mg GAE/g dried weight.

### 2.5. Quantification of Tannins

For the determination of tannins, the freeze-dried extracts (0.01 g) were dissolved in 50% methanol. The quantitative determination of tannins was performed according to the method described in the European Pharmacopoeia 8th edition [26]. Thus, for the determination of total polyphenols, 2 mL of extract (diluted 1:25) were treated with 1 mL of Folin–Ciocâlteu reagent, 10 mL of distilled water, and 29% Na_2_CO_3_ solution. The flask was left in the dark at room temperature for 30 min. Afterwards, the absorbance of the solution was measured at 760 nm (A1). To determine the content of polyphenols, which were not adsorbed on the hide powder, 10 mL of extract were added to a volumetric flask with 0.1 g of hide powder. The mixture was shaken for sixty minutes and then filtered. Finally, 2 mL of this solution were used for the total polyphenols determination (A2). The total tannin content (TTC) was determined by comparing the absorbance difference (A1–A2) to a pyrogallol solution representing an external standard. The final TTC was expressed as % of pyrogallol/vegetal material.

### 2.6. Antioxidant In Vitro Assays

#### 2.6.1. Determination of DPPH Radical Scavenging Activity

The free radical scavenging activity was determined according to a previously described method with slight modifications [27]. Different concentrations of the extract solutions (50 µL freeze dried extract in 50% methanol) and methanolic solution of 0.1 mM DPPH (200 µL) were incubated in darkness at room temperature for 30 min. The absorbance of each reaction mixture was read at 517 nm against the corresponding blank with a microplate reader (Epoch, BioTek, Winooski, VT, USA). The inhibition capacity was calculated using the following equation:

(1) $$\mathrm{inhibition}\;\left(\%\right)=\frac{A_{c}-A_{s}}{A_{c}}\times 100$$

where Ac is the absorbance of the control solution determined in the same conditions and As is the absorbance of the sample [28].

The concentration of extract or pyrogallol solution responsible for 50% inhibition of the DPPH radical (IC50) was determined from the nonlinear regression plot of inhibition percentage against the logarithms of concentrations.

#### 2.6.2. Determination of ABTS Free Radical-Scavenging Activity

Radical scavenging activity was measured as previously described with slight modifications [29]. Briefly, 50 µL of the extract solutions (freeze dried extract in 50% methanol) and 200 µL of ABTS methanolic solution (10 mM) were incubated at room temperature. The absorbance of each sample was read at 734 nm with a microplate reader. The inhibition capacity was calculated using the following equation:

(2) $$\mathrm{inhibition}\;\left(\%\right)=\frac{A_{c}-A_{s}}{A_{c}}\times 100$$

where Ac is the absorbance of the control solution determined in the same conditions and As is the absorbance of the sample.

### 2.7. Antibacterial Activity

The antibacterial activity of the freeze-dried extracts was quantified by determining the minimum inhibitory concentrations (MICs), using the microdilution method as previously described [30]. Briefly, the freeze-dried extracts were redissolved in 5% DMSO (achieving a concentration of 10 mg/mL) and added (200 µL) to the wells of the first column of a 96-well plate. The tested solutions were then binary diluted with sterile distilled water. Bacterial suspensions of each strain were prepared by adding 10 µL of inoculum in 9990 µL of Muller–Hinton broth 2X. A total of 100 µL of the bacterial suspensions were added to each well. For each tested substance and bacterial strain, the method was performed three times. Both the tested extracts and each bacterial strain suspension were added in separate wells as negative and positive controls, respectively. Additionally, a row of the microdilution plate was used to screen the potential antibacterial activity of the DMSO in the mixture. Finally, the microdilution plates were left in an incubator at 37 °C for 24 h. The MICs were considered in the first wells where no bacterial growth was observed (optical evaluation).

### 2.8. Statistical Analysis

All the determinations were performed in triplicate. The statistical analysis was performed using GraphPad Prism 8. The significance level was chosen before performing the statistical analysis (α = 0.05). First, the Kolmogorov–Smirnov normality test was used to assess if the data were taken from a Gaussian distribution. The F test was then used to compare the variance differences between the 2 groups. The correct *t*-test was chosen accordingly, comparing the means of the two data series. A *p* value less than 0.05 was considered significant.

## 3. Results and Discussions

### 3.1. Design of Experiments

When quantitative and qualitative factors are studied in a D-optimal design of experiments, the selection of experimental runs included in the design matrix is critical and should be performed employing scientific methods. In the present case, two statistical parameters were taken into account: condition number and G-efficiency [31].

The condition number assesses the sphericity of a design and is computed for an extended design matrix. Briefly, it represents the ratio of the largest and smallest values of the variability matrix. The ideal condition number value is 1, representing an orthogonal design. For optimization designs with multilevel factors, as in this case, the condition number value could increase considerably; therefore, a DoE with a condition number <8 is considered statistically efficient. For a high quality, reliable D-optimal DoE, a G-efficiency above 50% is recommended [22].

The condition number for the DoE had a value of 3.84 and a G-efficiency of 57%, values which describe a highly reliable statistical model [22]. The studied response was the TPC, assayed for each individual sample.

The results were introduced into the design matrix and the fitting of the experimental data was carried out using multiple linear regression (MLR). The model fitting was evaluated using the standard, most reliable statistical parameters: R2 (goodness of fit), which describes the fraction of the response variation explained by the model; Q2 (goodness of prediction), which estimates the prediction capacities of the model; and ANOVA test and model reproducibility, which are calculated and represented based strictly on the replicates specified in the design matrix [32].

A good fitting is represented by high values of the model performance indicators, as close as possible to value of 1. Moreover, for a valid model, the difference between R2 and Q2 should be no more than 0.2–0.3, as higher differences indicate an inappropriately selected model. Reproducibility should be well over 0.5 [33].

The summary of fit and the statistical parameters were calculated based on the experimental design data.

**Figure 1:**
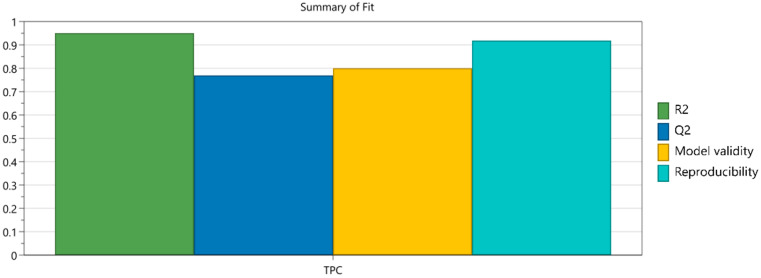
Summary of fit for DoE model.

The results confirmed that the developed model was statistically robust, indicating a significant influence of the factors on the responses and that the model has no lack of fit (Figure 1). Similar statistical results have been registered in other studies where the DoE has been applied for optimization purposes [34].

### 3.2. Effects of Process Variables on the Extracted TPC

**Figure 2:**
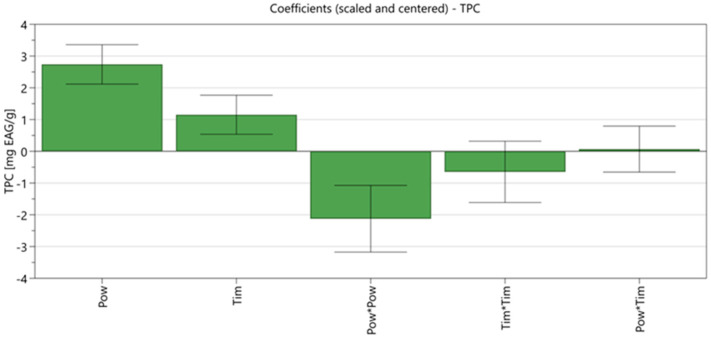
Scaled and centered coefficient plot.

The regression coefficients were automatically established for the studied variables based on the DoE model. Figure 2 presents the scaled and centered coefficient plots that highlight the influence of the extraction parameters over the extracted TPC content for water and EtOH 70% extract, respectively.

All parameters studied had a statistically significant influence. Irradiation time was the least influential parameter, whereas the irradiation power had a positive influence; increasing the microwave power increased the TPC of extracts.

The interaction between parameters is further discussed here. Power × power and time × time are quadratic interactions, meaning that the influence exerted on TPC solely by time or power is not linear. The interaction exerted by the two factors combined (time × power) is positive, meaning that an increase in the two would enhance their effect on TPC. It is important to note that such interactions can only be assessed using experimental designs [32].

### 3.3. Design Space and Process Optimization

**Figure 3:**
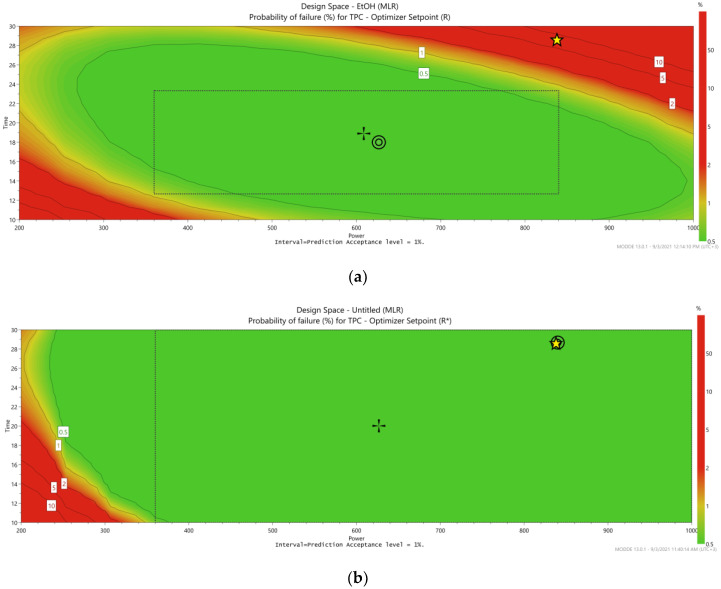
Design space for EtOH 70% extract (**a**) and water extract (**b**).

The previously described approach provided a comprehensive understanding of the variables’ influence over the extraction process. Next, the optimization function of MODDE software was used to generate a design space by introducing the desired TPC values in order to maximize the extraction capacities (Figure 3).

The desired values that were introduced to the MODDE software included: a TPC minimum of 25 mg GAE/g oak bark and target of 27 mg GAE/g oak bark for the water extract, and a minimum 30 mg GAE/g oak bark and target of 31.5 mg GAE/g oak bark for the 70% EtOH extract.

The desired process parameters were set as follows: irradiation time as short as possible and microwave power low as possible. Given these parameters, the optimization feature of the optimization software generated the following extraction parameters: for 70% EtOH extract, an irradiation time of 18 min at a microwave power of 650 W, and for water extract, an irradiation time of 30 min at 850 W.

The experimentally obtained TPC was 26.19 ± 0.29 mg GAE/g oak bark for the water extract and 31.27 ± 1.80 mg GAE/g oak bark for the 70% EtOH extract.

### 3.4. Total Polyphenolic and Tannin Content

The TPCs of the aqueous and ethanolic extracts were determined after the freeze-drying process and reconstitution, considering the absorption of the samples after the Folin–Ciocâlteu reaction and the calibration curve of the gallic acid standards: y = 11.767x + 0.2737, R^2^ = 0.9984. The results were expressed as mg GAE/g dry weight, and they are presented in Table 2.

As shown in Table 2, the QCE variant had a significantly higher level of total polyphenols (*p* = 0.0015), suggesting that the 70% ethanol–water mixture was a better solvent for the extraction of polyphenolic compounds from the *Q. cerris* bark. A previous study also indicated the presence of polyphenols in the bark of *Q. cerris*, presenting relatively similar values of TPC compared to our results [15]. Moreover, data in the literature suggest that using mixtures of solvents, such as our combination, may result in higher polyphenolic yields when using microwave assisted extraction because of a greater solubility of the target compounds and a better penetrability of the vegetal material [35,36].

In contrast with the results regarding the polyphenol content, the total tannin content was slightly higher in the QCA variant; however, the difference was not significant. This indicates that water and the ethanol–water mixture had a similar efficiency regarding the extraction of tannins from the hard vegetal matrix of the bark

### 3.5. Antioxidant Activity

**Figure 4:**
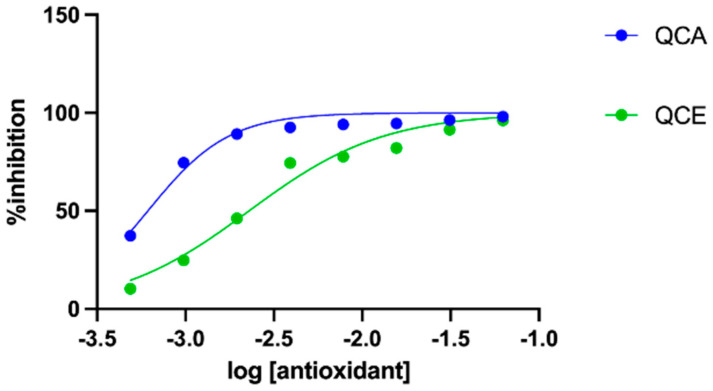
Logarithmic curves of antioxidant activity of QC extracts (n = 3).

Antioxidant activity was determined using the DPPH and ABTS methods, and results are presented in Table 2. The DPPH assay showed that the aqueous extract had significantly higher antioxidant activity compared with the ethanolic extracts, which is likely related to the tannin content (although the concentration of tannins was not significantly different between the two extracts, and the aqueous extract contained a higher concentration than the ethanolic extract). As can be seen in Figure 4, the two extracts had different interactions with the DPPH radical. The IC_50_ of pyrogallol was 6.67 ± 0.47, which indicates a high antioxidant potential of the tested extracts. As can be seen in Table 2, the DPPH assay results could not be correlated with the total polyphenolic content. Such discrepancies could be attributed to the fact that different phenol compounds react differently with Folin–Ciocâlteu reagent; in addition, not all phenolic compounds are potent radical scavengers, nor do they all have the same matrix effect [37]. The ABTS^•^+ scavenging assay, however, did not highlight any differences between the two extracts. It has been previously reported that, depending on the matrix of the herbal drug, DPPH and ABTS results sometimes cannot be correlated [38].

### 3.6. Antibacterial Activity

The antibacterial activity of the redissolved freeze-dried extracts was tested on the previously mentioned bacterial strains by determining the MICs of the experimental variants. As shown in Table 3, both tested extracts exhibited improved activity against the Gram-positive bacterial strains in comparison to the Gram-negative strains, especially QCE, which inhibited both *S. aureus* and MRSA at an MIC of 0.625 mg/mL. Similarly, QCE had improved activity against the Gram-negative bacterial strains compared to QCA, with the exception of *K. pneumoniae* for which both extracts exhibited the same MIC. Additionally, the DMSO used for redissolving the freeze-dried extracts had no effect against any of the tested bacterial strains; thus, the registered antibacterial effects were caused by the presence of the tested extracts. Previous studies have also indicated the antibacterial potential of extracts obtained from *Quercus* spp. barks. This activity hints towards the presence of polyphenols in the mentioned extracts [5,6,39,40].

## 4. Conclusions

The results revealed that aqueous and hydroalcoholic extracts have distinct optimal extraction conditions (30 min at 850 W for the aqueous extracts and 18 min at 650 W for the hydroalcoholic extracts). The hydroalcoholic bark extract had a larger yield of total polyphenols and a lower quantity of tannins when compared to the aqueous extract, and both extracts had considerable antioxidant activity. Furthermore, the extracts showed antibacterial activity against the tested bacterial strains, particularly against the Gram-positive strains and *Klebsiella pneumoniae*, with the hydroalcoholic extracts being more effective overall. To summarize, the improved MAE is an effective approach for extracting phytochemical components from *Quercus cerris* bark with possible biological effects.

## Figures and Tables

**Table 1:** Experimental parameters set by DoE for the optimization of the extraction method.

Exp No	Exp Name	Run Order	Power (W)	Time (min)
1	N1	11	200	10
2	N2	13	1000	10
3	N3	2	1000	10
4	N4	4	500	10
5	N5	10	200	30
6	N6	12	1000	30
7	N7	3	1000	30
8	N8	1	500	30
9	N9	14	200	20
10	N10	9	1000	20
11	N11	6	500	20
12	N12	7	500	20
13	N13	8	500	20
14	N14	5	500	20

**Table 2:** Total polyphenolic content (TPC), total tannin content (TTC), and antioxidant activity.

Sample	TPC (mg GAE/g d.w.)	IC_50_ DPPH (µg/mL)	IC_50_ ABTS (µg/mL)	TTC (%)
QCA	382.26 ± 0.97 ^a^	2.446 ± 0.24 ^a^	6.211 ± 0.51	49.14 ± 1.36 ^a^
QCE	403.73 ± 7.35 ^b^	6.92 ± 0.39 ^b^	6.19 ± 0.35	45.68 ± 1.75 ^a^

Note: d.w.—dry weight. Different superscript letters (a, b) in the same column mean statistically significant differences at *p* < 0.05. QCA—*Q. cerris* aqueous bark extracts; QCE—*Q. cerris* ethanolic bark extracts.

**Table 3:** Antibacterial activity (MIC in mg/mL) of the *Q. cerris* bark extracts.

Bacterial Strain	MIC (mg/mL)	
	QCA	QCE
Gram-positive bacteria		
*Staphylococcus aureus* ATCC 25923	0.625	0.625
Methicillin-resistant *Staphylococcus* *aureus* ATCC 43300	1.25	0.625
Gram-negative bacteria		
*Escherichia coli* ATCC 25922	>5	2.5
*Klebsiella pneumoniae* ATCC 13883	1.25	1.25
*Pseudomonas aeruginosa* ATCC 27853	>5	5

QCA—*Q. cerris* aqueous bark extracts; QCE—*Q. cerris* ethanolic bark extracts.

## Data Availability

Not applicable.
